# Differential Localization and Functional Specialization of *parS* Centromere-Like Sites in *repABC* Replicons of *Alphaproteobacteria*

**DOI:** 10.1128/aem.00207-22

**Published:** 2022-04-07

**Authors:** Jakub Czarnecki, Elvira Chapkauskaitse, Julia Bos, Dorota Sentkowska, Paweł Wawrzyniak, Agnieszka Wyszyńska, Magdalena Szuplewska, Dariusz Bartosik

**Affiliations:** a Department of Bacterial Genetics, Institute of Microbiology, Faculty of Biology, University of Warsaw, Warsaw, Poland; b Institut Pasteur, Université de Paris, CNRS, UMR3525, Unité Plasticité du Génome Bactérien, Département Génomes et Génétique, Paris, France; c Department of Microbial Biochemistry, Institute of Biochemistry and Biophysics, Polish Academy of Sciences, Warsaw, Poland; University of Tartu

**Keywords:** *Alphaproteobacteria*, partitioning system, plasmid evolution, plasmid segregation, repABC

## Abstract

Partitioning systems ensure the stable inheritance of bacterial low-copy-number replicons, such as chromosomes, chromids, and megaplasmids. These loci consist of two genes encoding partition proteins A and B, and at least one *parS* centromere-like sequence. In chromids and megaplasmids, partitioning systems are often located in the vicinity of replication systems. An extreme example of this co-localization are alphaproteobacterial *repABC* replicons, where the partition (*repAB*) and replication (*repC*) genes form a single operon, with *parS* sequences usually positioned in close proximity to these genes. In this study, we characterized a more complex *repABC* system found in Paracoccus aminophilus (*Rhodobacterales*) megaplasmid pAMI4 (438 kb). Besides the *repABC* operon with a single *parS* site, this replicon has a 2-kb non-coding locus positioned 11.5 kb downstream of *repC*, which contains three additional *parS* repeats (*3parS*). We demonstrated that *3parS* is bound by partition protein B *in vitro* and is essential for proper pAMI4 partitioning *in vivo*. In search of similar loci, we conducted a comparative analysis of *parS* distribution in other *repABC* replicons. This revealed different patterns of *parS* localization in *Rhodobacterales* and *Rhizobiales*. However, in both these taxonomic orders, *parS* sites are almost always located inside or close to the *repABC* operon. No other *3parS*-like loci were found in the closest relatives of pAMI4. Another evolutionarily-independent example of such a locus was identified as a conserved feature in chromosome 2 of Allorhizobium vitis and related replicons.

**IMPORTANCE** The *repABC* replication/partitioning loci are widespread in extrachromosomal replicons of *Alphaproteobacteria*. They are evolutionarily diverse, subject to multi-layer self-regulation, and are responsible for the maintenance of different types of replicons, such as plasmids (e.g., *Agrobacterium* pTi and pRi tumorigenic and rhizogenic plasmids), megaplasmids (e.g., *Sinorhizobium* pSymA and pSymB) and essential chromids (e.g., secondary chromosomes of *Agrobacterium*, Brucella and *Rhodobacter*). In this study, we functionally analyzed an atypical partition-related component of *repABC* systems, the *3parS* locus, found in the *P. aminophilus* megaplasmid pAMI4. We also identified *parS* centromere-like site distribution patterns in different groups of *repABC* replicons and found other unrelated *3parS*-like loci, which had been overlooked. Our findings raise questions concerning the biological reasons for differential *parS* distribution, which may reflect variations in *repABC* operon regulation as well as different replication and partition modes of replicons belonging to the *repABC* family.

## INTRODUCTION

Partition of most bacterial chromosomes and large low-copy-number extrachromosomal replicons, including megaplasmids and chromids (often called secondary chromosomes), is dependent on ParAB-*parS* partitioning systems. These systems consist of a *parAB* operon, encoding an ATPase (ParA) and a DNA-binding protein (ParB), as well as palindromic sequences analogous to eukaryotic centromeres (*parS*) (1, 2).

The action of this system is performed in two steps: (i) ParB binding to the replicated *parS* sequences and (ii) ParA-driven separation of the ParB-*parS* complexes toward the opposite cell poles. The detailed mechanism of ParB binding to *parS* and its spread to adjacent DNA has recently been described (3).

Megaplasmid and chromid partitioning modules are often encoded in the vicinity of modules involved in plasmid replication. The most intricate examples of this co-existence are the *repABC* replicons found exclusively in *Alphaproteobacteria.* In this unique group of replicons, a single operon *repABC* encodes the proteins involved in both plasmid partition (RepA and RepB – homologs of ParA and ParB) and replication (RepC plasmid replication initiator). The *repABC* modules also contain the origin of replication (*oriV*), located inside the *repC* gene, as well as single or multiple *parS* sites, found at different locations within the module (4–7). The regulation of *repABC* expression occurs at many levels, including negative self-regulation of operon transcription by the RepA and RepB proteins, and modulation of *repC* transcript translation by a small regulatory ctRNA (RepE), encoded between *repB* and *repC* (8).

The *repABC* modules are also commonly found in extrachromosomal replicons of *Paracoccus* spp. (*Rhodobacteraceae*, *Alphaproteobacteria*), including *P. aminophilus* JCM 7686 – a methylotrophic strain isolated in Japan from a sample of soil polluted with dimethylformamide (DMF) (9–11). The multi-replicon genome of this strain contains eight extrachromosomal replicons (10). One of these, megaplasmid pAMI4 (438 kb), carries a *repABC* system, that, besides one *parS* centromere-like site localized in the classic manner in the *repABC* operon, may employ three additional *parS* repeats within a long (2.5 kb), low GC, non-coding region (henceforth referred to as the *3parS* locus), located 11.5 kb downstream of *repC*. In this study, we demonstrated that these extra *parS* repeats are essential for the proper partitioning of pAMI4 and its stability in the host cell population. In addition, we conducted a bioinformatic analysis of a large set of *repABC* replicons from *Rhodobacterales* and *Rhizobiales*, which show different patterns of *parS* localization. Interestingly, we identified other *3parS*-like loci that are evolutionarily unrelated to that of pAMI4.

## RESULTS

### Structure of the pAMI4 *repABC* module.

**FIG 1:**
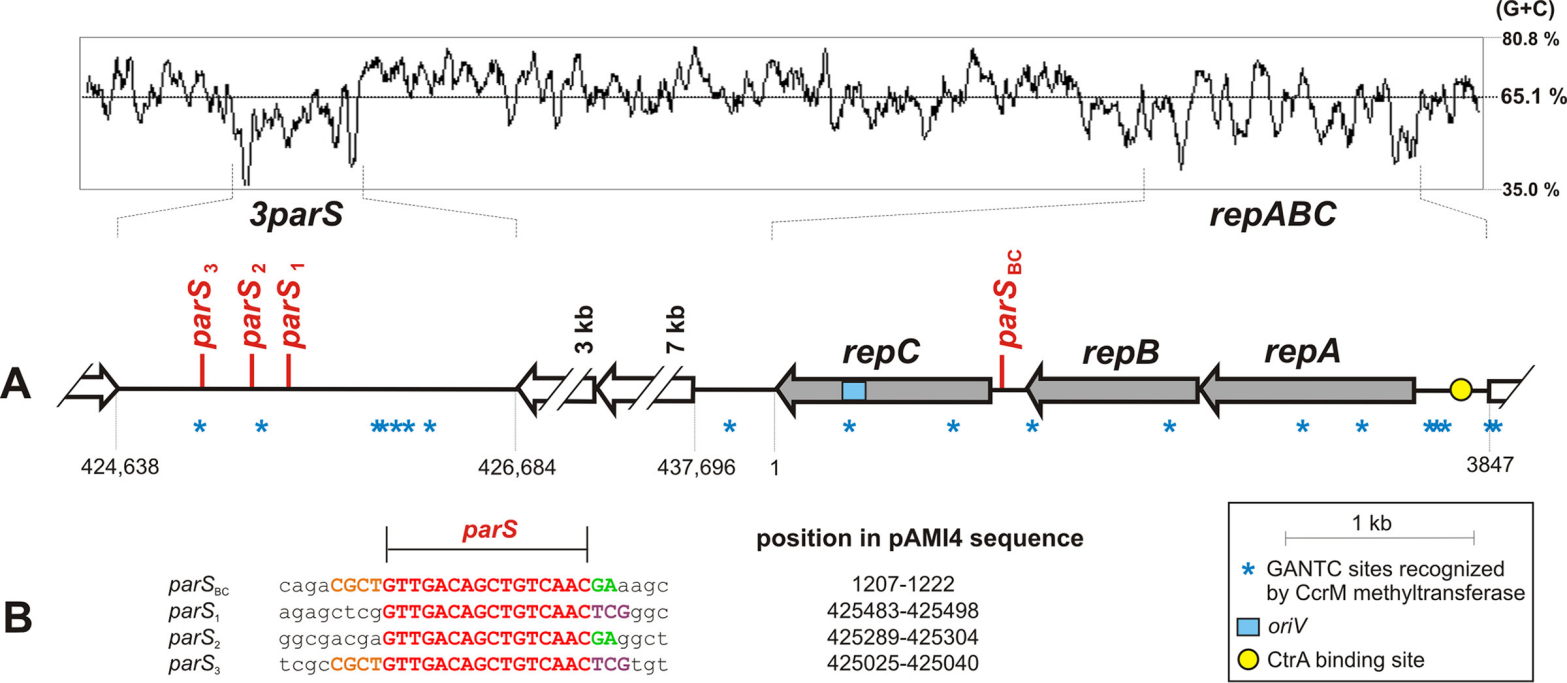
Distribution of *parS* sites in the pAMI4 genome. (A) Genetic organization of the DNA region containing *repABC* and the *3parS* locus. Numbers below the diagram correspond to nucleotide positions in the pAMI4 sequence deposited in the GenBank database (NC_022049). Genes of the *repABC* module are marked in gray. The GC profile of the analyzed pAMI4 sequence is shown in the top panel. (B) Comparison of the identified *parS* sites (in red) in their original sequence context. Conserved nucleotides are shown in upper case letters. The colors orange, green, and violet indicate different types of conserved neighboring sequences.

The pAMI4 *repABC* operon has a typical organization, with overlapping *repA* and *repB* genes (1-bp overlap) and a *repC* gene preceded by a 175-bp-long intergenic region (Fig. 1). This region contains a *parS* site (*parS*_BC_, 5′-GTTGACAGCTGTCAAC-3′), that matches the *parS* consensus sequence for *repABC* replicons, 5′-GTTNNCNGCNGNNAAC-3′, determined by Cevallos et al. (5).

The pAMI4 *repABC* operon carries a functional origin of replication as it was used for construction of a pAMI4 mini-derivative that replicates in *Paracoccus* spp. (10). Interestingly, 11.5 kb downstream of the *repC* gene, beyond two large open reading frames (ORFs) encoding hypothetical membrane proteins, we identified a long (2047 bp) intergenic region carrying three more *parS* repeats (*parS*_1-3_) (Fig. 1). These repeats are identical to *parS*_BC_, and some local sequence similarities are also found in their immediate vicinity (Fig. 1B). This *3parS* region lacks any remnant genes and its nucleotide sequence has a higher AT content (42.5%) than the pAMI4 sequence as a whole (35.8%) or the *oriV*-containing *repC* gene (37.3%).

Further analysis of the DNA region containing *repABC* and the *3parS* locus revealed the uneven distribution of GANTC sites recognized by the CcrM methyltransferase (Fig. 1) – a key epigenetic mediator in *Alphaproteobacteria* (12), which is equivalent to the gammaproteobacterial Dam methylase (13). This observation strongly suggests cell cycle dependent regulation of *repABC* operon transcription (three GANTC sites in the *repABC* promoter region) and an unknown type of regulation occurring in the *3parS* region (five GANTC sites ∼500 bp from *parS*_3_) (the mean distribution of GANTC sites in the pAMI4 genome is one per 1132 bp) (Fig. 1). It is noteworthy that the intergenic region upstream of *repABC* also contains a putative CtrA-binding site (5′-CTAAN_7_TTAAC-3′), with one nucleotide difference compared to the consensus CtrA-binding site of Caulobacter crescentus (14). CtrA is a global regulator of many cell cycle-dependent genes in *Alphaproteobacteria* and a negative regulator of replication initiation of the C. crescentus chromosome (14). The *ctrA* gene is also found in the *P. aminophilus* genome (GenBank acc. no. AGT08327). The presence of a CtrA-binding site within the promoter region of a *repABC* operon was previously observed in the pTi plasmid of Agrobacterium tumefaciens (15).

### Function of the *3parS* locus in pAMI4 partition.

To confirm that the *3parS* locus is involved in the partitioning of pAMI4 molecules, *in vitro* and *in vivo* analyses were performed to determine whether this region possesses the three basic properties typical for plasmid centromere-like sites: (i) expression of incompatibility behavior, (ii) interaction with the RepB protein, and (iii) the ability to ensure *in cis* stability of a plasmid in the presence of partition proteins. Furthermore, a Δ*3parS* mutant was obtained and the effect on pAMI4 stability and the positioning of a GFP-RepB_pAMI4_ fusion protein in the cells was investigated.

The *parS* sites are strong incompatibility determinants (*inc*) and therefore their introduction into the strain of origin may result in removal of the parental *parS*-containing plasmid from the bacterial cells (16). The entire *3parS* locus was cloned into vector pBBR1MCS-5, and the resulting plasmid was introduced into *P. aminophilus* JCM 7686R (Rif^r^) by triparental mating. All tested Gm^r^ Rif^r^ transconjugants carrying the autonomous form of pBBRGm-3parS had lost pAMI4, which was verified by PCR with pAMI4-specific primers. This confirmed that the *3parS* region exerts incompatibility toward pAMI4. At this stage, a *P. aminophilus* pAMI4-less strain, designated UW100, was obtained (lacking both pAMI4 and pBBRGm-3parS), for use as a convenient host for pAMI4-based mini-derivatives in subsequent experiments.

**FIG 2:**
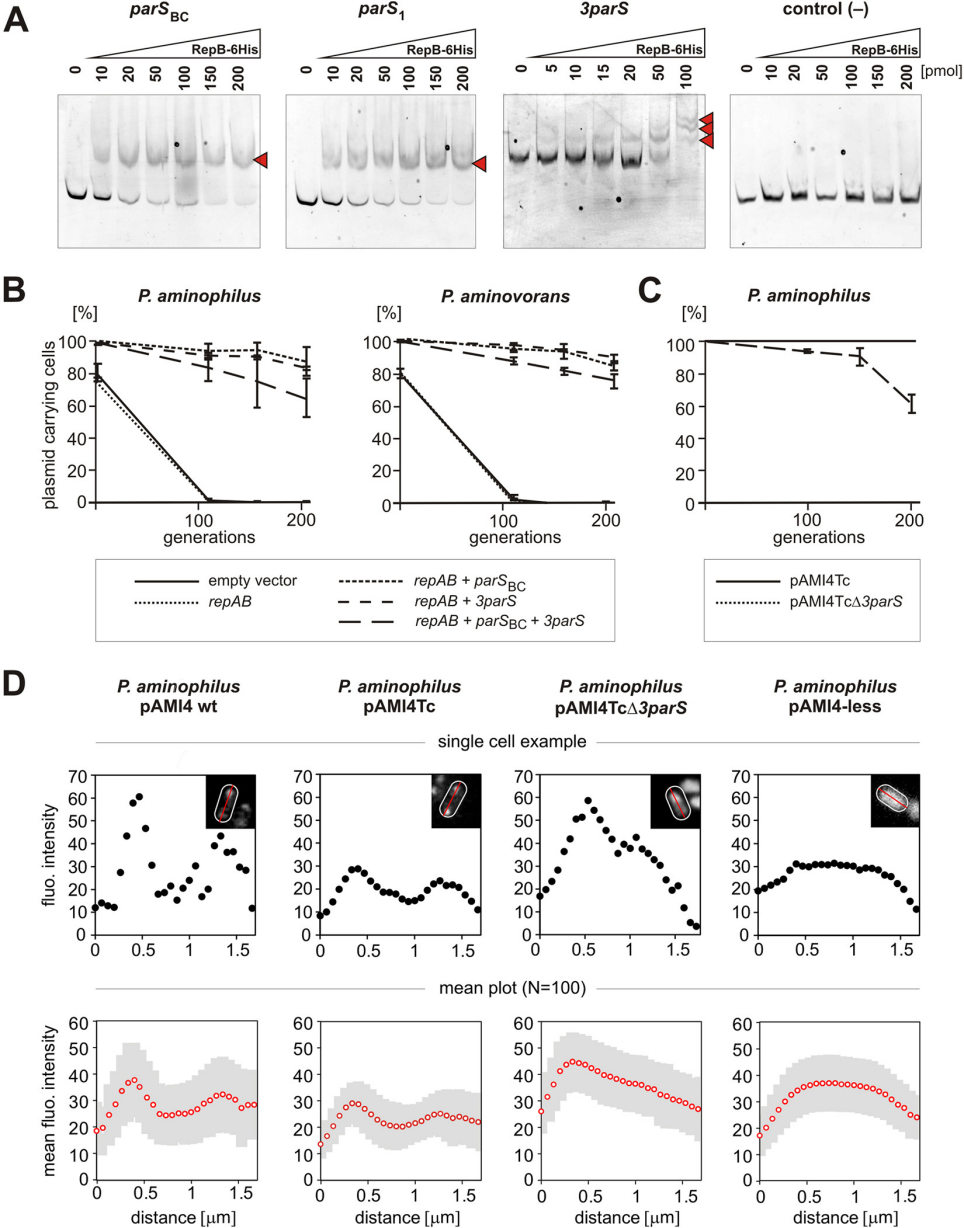
Functional analysis of the *3parS* locus. (A) Electrophoretic mobility shift assays of the 6His-RepB_pAMI4_ protein with pAMI4 *parS* sequences. The protein-DNA complexes are marked with red arrowheads. The tested DNA fragments of pAMI4, labeled with fluorescein, were incubated with increasing amounts of the 6His-RepB_pAMI4_ protein. The *par*S_1_, *par*S_2_ and *par*S_3_ sites showed the same pattern of 6His-RepB_pAMI4_ binding. Therefore, the result for only one of them (*parS*_1_) is presented. FAM-labeled DNA fragment of pBGS18 was used as a negative control. (B) Stability assays of test vector pABW3 (Km^r^) containing different combinations of the components of the pAMI4 partitioning system (*repAB*, *parS*_BC_ and the *3parS* locus). *P. aminophilus* UW100 and *P. aminovorans* JCM 7685R transformed with different pABW3 constructs were grown under nonselective conditions. Retention of plasmids was determined by the percentage of kanamycin resistant cells at each time interval (100 colonies tested by replica plating, the plotted values are the means from triplicate assays and standard deviations are shown). (C) Stability assays of pAMI4Tc (control) and pAMI4TcΔ*3parS* in its natural host *P. aminophilus* during growth under non-selective conditions. Retention of plasmids was determined by the percentage of tetracycline resistant cells at each time interval (100 colonies tested by replica plating, the plotted values are the means from triplicate assays and standard deviations are shown). (D) Measurement of GFP-RepB_pAMI4_ fluorescence intensity profiles in various *P. aminophilus* strains with pJC43-GFP-RepB cultured to mid-exponential phase in LB with gentamicin and taurine. From left to right: *P. aminophilus* JCM 7686R carrying pAMI4 wt, pAMI4Tc or pAMI4Tc*Δ3parS*, and *P. aminophilus* UW100 (pAMI4-less). The top row of plots show the typical patterns of fluorescence in cells expressing GFP-RepB_pAMI4_ (black dots). The fluorescence intensity signal was measured along the bacterial longitudinal axis (red line from one pole of the cell (outlined in white) to the other pole (inset pictures)) using ImageJ (Fiji) software. The bottom row of plots show the mean fluorescence intensity (empty red dots) and standard deviation (gray shaded area) over the length of the cells (*N* = 100). Enlarged microscopy images of *P. aminophilus* cells expressing GFP-RepBpAMI4 are presented in Fig. S1 in the supplemental material.

To confirm that *3parS* interacts with RepB_pAMI4_, an *in vitro* electrophoretic mobility shift assay (EMSA) was performed. The pAMI4 *repB* gene was cloned into vector pET28b(+) in order to express a 6×His-RepB_pAMI4_ fusion protein. The purified protein was used in EMSAs with DNA fragments containing (i) *parS*_BC_ of the *repABC* module (positive control), (ii) individual *parS* sites from the *3parS* locus – *parS*_1_, *parS*_2_, *parS*_3_, (iii) the entire *3parS* region, and (iv) a DNA fragment from vector pBGS18 (negative control). The 6×His-RepB_pAMI4_ protein was found to specifically bind *parS*_BC_ and the entire *3parS* region, as well as each of the single *parS*_1-3_ sequences. In the case of the fragment comprising the entire *3parS* region, three complexes with different electrophoretic mobilities were formed in a protein concentration-dependent manner, most probably representing the binding of 6×His-RepB_pAMI4_ to one, two, or three *parS* sites within *3parS* (Fig. 2A).

The stabilizing effect of the pAMI4 partitioning system on a test plasmid was examined in its natural host *P. aminophilus* strain UW100 and in a heterologous host *P. aminovorans* JCM 7685R (17). Several derivatives of vector pABW3 (Km^r^; unstable in *Paracoccus* spp.) (4) were constructed for this analysis, containing (i) the *repAB* genes alone, (ii) *repAB* with its cognate *parS*_BC_, (iii) *repAB* with the *3parS* region, and (iv) *repAB* with *parS*_BC_ and *3parS*. The obtained constructs were introduced into both strains by triparental mating. The stability of the different plasmids was tested by monitoring the proportion of kanamycin resistant cells during growth in liquid culture without antibiotic selection (Fig. 2B).

The results showed that *3parS* fully compensates for the lack of *parS*_BC_ in both bacterial hosts. However, the presence of *3parS* and *parS*_BC_ in the same plasmid was found to decrease the stabilizing effect compared to plasmids carrying only *3parS* or only *parS*_BC_ (Fig. 2B). A similar effect was observed in the case of the *parS* sequences of pSymA of Sinorhizobium meliloti 1021, a *repABC* replicon with six *parS* repeats upstream of *repA*. The first *parS* (counting from *repA*) stabilized a test plasmid carrying the *repAB* genes of pSymA, while addition of the other five sequences destabilized the system (18). It should be noted that results obtained in assays performed using small plasmids do not necessarily reflect the function of the tested *parS* sites in large natural replicons.

To test the function of *3parS* in its original context, a Δ*3parS* strain was constructed in a pAMI4-derivative carrying a tetracycline resistance cassette (pAMI4Tc). The stability of the mutated plasmid was tracked during growth in LB medium for up to ∼200 generations, by monitoring the fraction of tetracycline resistant cells. The deletion of *3parS* caused a significant drop in pAMI4 stability. At the final checkpoint, less than 70% of the cell population carried pAMI4TcΔ*3parS*, compared to 100% stability of pAMI4Tc (Fig. 2C).

Finally, to investigate the influence of *3parS* on the formation of RepB_pAMI4_ foci, a plasmid for taurine-dependent expression of a GFP-RepB_pAMI4_ fusion protein was constructed and introduced into *P. aminophilus* JCM 7686R carrying pAMI4 (wt), pAMI4Tc or pAMI4TcΔ*3parS*, or strain UW100. Two green fluorescent foci were formed in the control strains *P. aminophilus* JCM 7686R carrying pAMI4 (wt) or pAMI4Tc, while in the pAMI4TcΔ*3parS* strain the GFP was much more dispersed, with a higher fluorescent signal on only one side of the cell. In the pAMI4-less strain UW100, the fluorescence was evenly distributed throughout the cell (Fig. 2D and Fig. S1 in the supplemental material).

### Distribution of *parS* sites in *repABC* replicons.

To our knowledge, no other *repABC* replicon carrying an additional locus involved in partition has been described previously. Therefore, to identify loci similar to *3parS* and to uncover its evolutionary origin, we analyzed the distribution of *parS* sites in diverse *repABC* replicons available in the NCBI database using two different data sets. The first data set, comprised of 115 complete plasmid genomes, represented the diversity of *repABC* replicons found in *Rhodobacterales* and *Rhizobiales* (*Alphaproteobacteria*) (Table S1 in the supplemental material). The analysis of this data set was to determine the variability of *parS* localization on a larger evolutionary scale without losing information about *parS* sites located distantly from the *repABC* operon (only complete plasmid sequences are included in the data set). The second data set represented the 100 closest relatives of pAMI4 (according to RepB similarity) identified in NCBI GenBank and is comprised mainly of shotgun sequencing contigs (Table S2).

**FIG 3:**
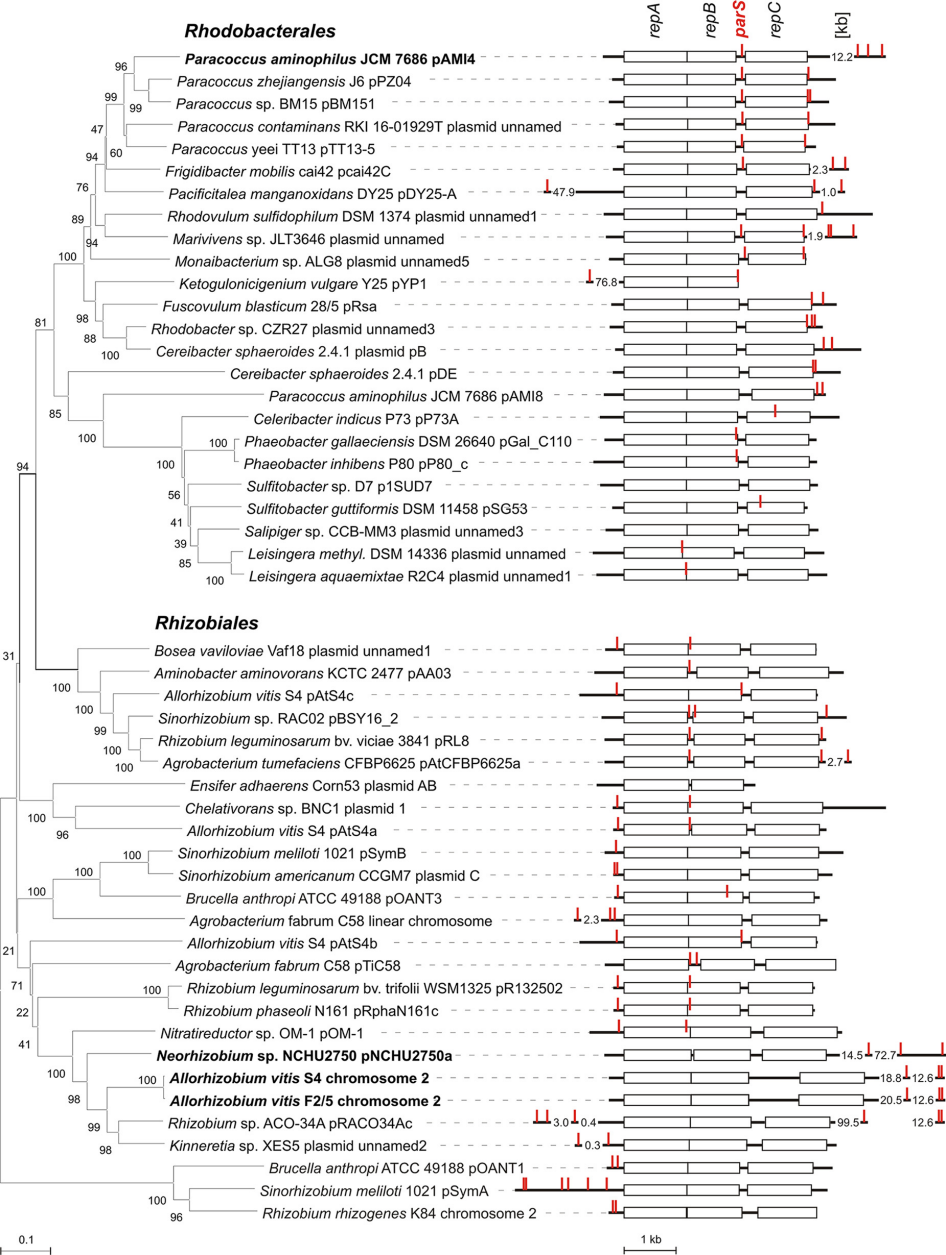
Distribution of putative *parS* sequences within *repABC* replicons from *Rhodobacterales* and *Rhizobiales*. Fifty representative replicons from Data set 1 (Table S1 in the supplemental material) are presented. In the left-hand panel, a neighbor joining tree of RepA-RepB concatenated protein sequences is shown. In the right-hand panel, the identified *parS* sequences are marked in red on diagrams of the *repABC* modules. These show the *repA*, *repB*, and *repC* genes (rectangles) and intergenic regions upstream of *repA*, between *repA* and *repB*, between *repB* and *repC*, and downstream of *repC* (bold lines). The intergenic/intragenic localization of putative *parS* sequences located outside the *repABC* modules (following the numbers giving the distance from the *repABC* module in kb) is not indicated. In some cases, putative *parS* sequences and/or the *repC* gene were absent. Replicons carrying long intergenic loci with *parS* repeats are in bold.

These analyses identified different *parS* localization patterns in *Rhodobacterales* and *Rhizobiales*. In *Rhodobacterales*, the *parS* sequences tend to be localized in the intergenic region between *repB* and *repC*, and directly downstream of *repC* (Fig. 3, Fig. S2 in the supplemental material). Multiplication of the *parS* sequences located downstream of *repC* is frequently observed, with up to four *parS* repeats found in this region. These sequences are located not only in the intergenic region directly downstream of *repC*, but also within the following ORFs and intergenic regions. The interval between the end of the intergenic region downstream of *repC* and the more distant *parS* sequences is typically between ∼0.5 and ∼4 kb, with pAMI4 being an exception at 12 kb (Fig. 3). Interestingly, few putative *parS* sequences were identified at locations very distant (tens of kb) from *repABC*. However, in *Rhodobacterales* we did not find any long non-coding loci with multiple *parS* site*s* resembling *3parS*.

**FIG 4:**
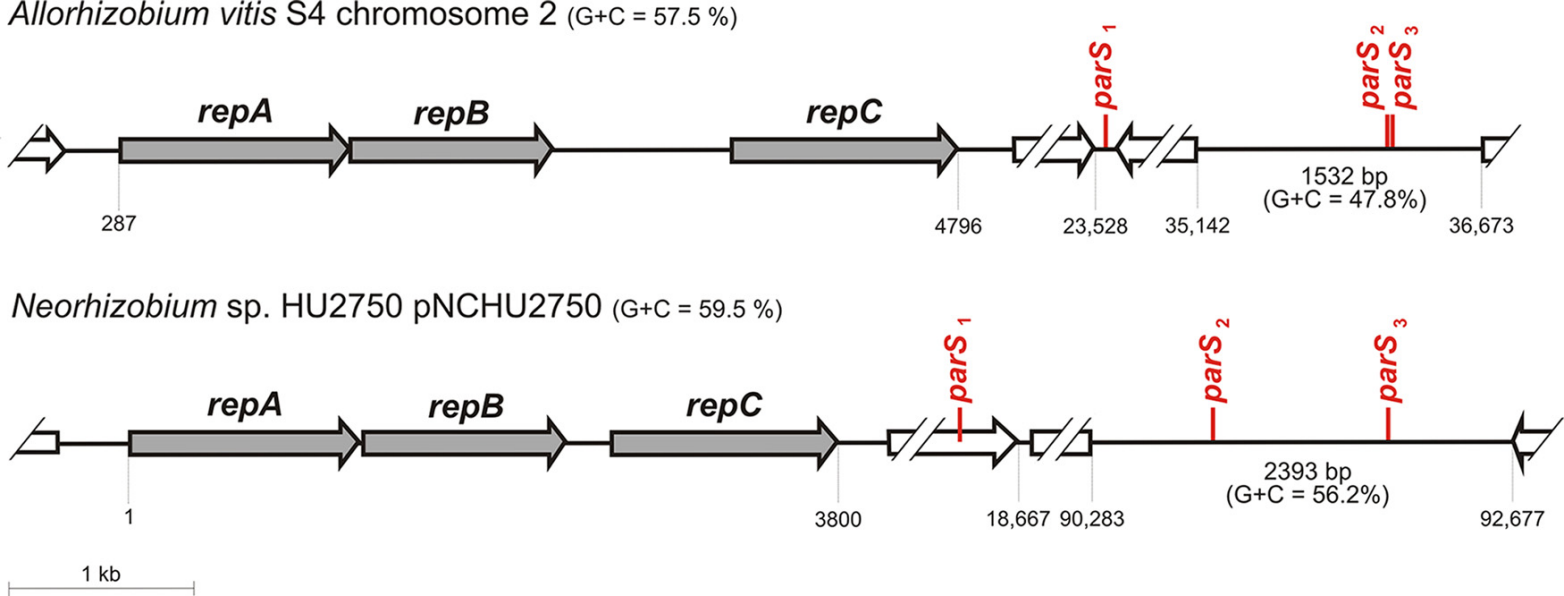
Distribution of putative *parS* sites in Allorhizobium vitis S4 chromosome 2 (NC_011988.1) and *Neorhizobium* sp. HU2750 plasmid pNCHU2750 (CP030828.1).

In *Rhizobiales*, the *parS* sequences tend to be localized in the intergenic region directly upstream of *repA*, and in the intergenic region between *repA* and *repB* (if these genes do not overlap), or at the 5′ end of *repB* (Fig. 3). There are some exceptions to this pattern, with the most interesting example present in chromosome 2 of Allorhizobium vitis S4 and its close relatives. These replicons do not have any *parS* sites located in the *repABC* operon. However, they possess a long (1.5-2.4 kb) non-coding AT-rich region located far downstream of *repC* (30-90 kb), containing two *parS* repeats (Fig. 4). This region resembles *3parS*, but it has an independent evolutionary history. Interestingly, all replicons carrying this *3parS*-like region, have an additional degenerate *parS* (1 nt different from other *parS* sites in the same replicon) located 15–20 kb downstream of *repC* in a short intergenic region (∼100 bp) or within a gene (Fig. 4, Table S2 in the supplemental material).

## DISCUSSION

Long inter-ORF regions are rare in bacteria and when they occur they often display regulatory functions. In this study, we identified and characterized such a region in *P. aminophilus* megaplasmid pAMI4. This locus, referred to as *3parS*, carries three functional *parS* centromere-like sites recognized by the RepB_pAMI4_ protein, which are essential for pAMI4 stability. Additional unusual features of *3parS* (high % AT compared to the whole plasmid and proximity to a region rich in GANTC sites, which are methylated by the CcrM methyltransferase, an important player in *Alphaproteobacteria* cell cycle regulation) indicate that this locus may be involved in other uncharacterized layers of pAMI4 maintenance regulation.

The role of the three *parS* locus is not immediately evident, as the *repABC* operon of pAMI4 contains another *parS* site, which could mediate the plasmid’s partition. However, we demonstrated that *3parS* is required for proper maintenance of this plasmid. The single *parS* site located between *repB* and *repC* is not sufficient to act as a focus for RepB_pAMI4_ nor to sustain full stability of pAMI4 under the tested conditions. This indicates possible specialization of the *parS* sites into those that play a leading role in partition (the sites located in the *3parS* region) and those performing another function (the site between *repB* and *repC*). Indeed, the latter *parS* site is located in a region where a gene encoding ctRNA RepE, a negative regulator of RepC translation, was identified in other *repABC* replicons (5). Thus, this site may participate in RepB-dependent regulation of RepE transcription, which might be a specific mechanism to modulate RepC expression.

Similar *parS* specialization might be common in other *repABC* replicons of *Rhodobacterales*. They frequently have the *parS* site in the *repB-repC* intergenic region, but also possess multiple *parS* repeats (two to four) located up to 4 kb downstream of *repC* (Fig. 3, Fig. S2 in the supplemental material). The pAMI4 *3parS* seems to have evolved from these latter sites, which were moved away from *repC* by an insertion. It is impossible to state whether such a new configuration was positively selected and conserved, as we lack information about the *repABC* systems that are very closely related to that of pAMI4 (the closest relatives of RepC_pAMI4_ in GenBank NCBI share only around 66% aa identity).

Interestingly, the *parS* sites located downstream of *repC* are found not only in the intergenic region directly after this gene. Many of them are located in subsequent intergenic regions or within ORFs (Fig. 3, Fig. S2 in the supplemental material). The most intriguing example of *parS* sequences located within ORFs are those identified in *cas* genes (*cas3* – encoding ATP-dependent single-strand DNA translocase/helicase that degrades DNA as part of CRISPR based immunity; *casA* and *casB* – encoding CRISPR-associated proteins of the multi-subunit ribonucleoprotein Cascade complex (19)). This arrangement was identified in six contigs originating from six different species (Marinibacterium profundimaris, *Frigidobacter mobilis*, *Tabrizicola* sp. YIM 78059, *Ruegeria intermedia*, Jhaorihella thermophila and *Paenimaribius caenipelagi*; the contig numbers may be found in Tables S1 and S2), and seems to have evolved several times, as judged by RepB- and Cas3-based phylogenetic trees (compare Fig. S2 and Fig. S3 in the supplemental material). We hypothesize that as the presence of multiple *parS* sites located downstream of *repC* may be advantageous for *repABC* replicons of *Rhodobacterales*, they are positively selected even when present within coding sequences (they may arise in this location *de novo* or they may be present in incoming genes that already contain such sequences).

We observed another pattern of *parS* distribution in *Rhizobiales*, with *parS* sites located upstream of *repA* and in the *repA/repB* intergenic region, or at the 5′ end of *repB.* Specialized roles for *parS* sites at such locations have been proposed and tested previously, i.e., transcription regulation of the *repABC* operon for sequences located upstream of *repA* and transcription regulation of *repC* only for sequences located in the *repA/repB* region (5). It is noteworthy that *repABC* replicons of *Rhizobiales* are often large (>1 Mb) and fulfill important biological roles for their hosts, e.g., symbiotic megaplasmids, essential chromids. Especially in these largest *repABC* replicons (pSymA, Rhizobium rhizogenes K84 chromosome 2, Agrobacterium tumefaciens chromosome 2), we observed multiplication of *parS* upstream of *repA* and the absence of the *parS* site located in the *repA/repB* region, which may reflect specific regulatory issues important for the maintenance of such large DNA molecules. Interestingly, in this group of replicons, we identified a locus which resembles *3parS* of pAMI4. It is a non-coding, AT-rich region containing two *parS* repeats located 30–90 kb downstream of *repC*, which is found in Allorhizobium vitis S4 chromosome 2 and related replicons. As the closest relatives of these replicons carry multiple *parS* sites upstream of *repA*, as do most *repABC* replicons in *Rhizobiaceae*, we suppose that this locus could have arisen by translocation of the *repA* upstream region to a position far downstream of *repC*.

The reasons for the different patterns of *parS* localization in *repABC* replicons of *Rhodobacterales* and *Rhizobiales* remain unclear. However, they may reflect differences in the features of these replicons in these two taxonomic orders. In *Rhodobacterales*, the *repABC* replicons appear to be smaller and less domesticated, while they are larger and better domesticated in *Rhizobiales* (and thus have more regulatory mechanisms to synchronize their replication and partition with the cell cycle) (20).

The *3parS* and similar loci described in this study may only be interesting exceptions from the general *parS* localization patterns typical for *Rhodobacterales* and *Rhizobiales*. Nevertheless, their specific features (low % GC, CcrM binding sites in the case of *3parS* of pAMI4) and evolutionary conservation (the case of *A. vitis* S4 chromosome 2 and its relatives) may lead to speculation that moving the *parS* function further from the *repABC* region is important for a specific mode of replication and partition, and regulation of these processes.

## MATERIALS AND METHODS

### Bacterial strains cultivation.

*Paracoccus* spp. were grown at 30°C on LB (or LA) supplemented with antibiotics (rifampicin, 50 μg/mL; kanamycin, 50 μg/mL; gentamicin, 40 μg/mL; tetracycline, 2 μg/mL), sucrose (11% wt/vol) or taurine (20 mM), as required. E. coli was grown at 37°C in LB (or LA) medium, supplemented with antibiotics (kanamycin 50 μg/mL; gentamicin 40 μg/mL; tetracycline, 20 μg/mL) and diaminopimelate (DAP) (0.3 mM), as required.

### Genetic manipulations.

Routine DNA manipulation procedures were performed according to standard methods (21). Some plasmids were constructed by Gibson assembly (22). E. coli plasmid DNA was isolated using a Plasmid Mini isolation kit (A&A Biotechnology). *P. aminophilus* plasmid DNA was isolated using an alkaline lysis procedure (21). Bacterial genomic DNA was isolated using a GeneMATRIX Bacterial & Yeast Genomic DNA purification kit (EurX). Plasmid DNA was introduced into E. coli cells by the chemical transformation method of Kushner (23) and into *Paracoccus* spp. cells by bi- and triparental mating procedures (11). Genetic modifications of pAMI4 were performed by a gene replacement technique adapted to *P. aminophilus* (11, 24).

### Bacterial strains used in this study.

E. coli DH5α (21) was used as a standard cloning strain and as a donor for triparental mating. E. coli β2163 (*dapA*) (25) was used as a cloning strain for *ori* R6K plasmid pDS132 and as a donor for biparental mating for gene replacement in *P. aminophilus*. E. coli BL21 (26) was used as a strain for 6×His-RepB_pAMI4_ recombinant protein overexpression. *P. aminophilus* JCM 7686R (27) was used as a strain carrying the wild type pAMI4 megaplasmid. *P. aminovorans* JCM 7685R (17) was used as a heterologous host in plasmid stability assays. *P. aminophilus* pAMI4Tc was constructed based on *P. aminophilus* JCM 7686R by inserting a tetracycline resistance gene (*tetC*) between 383337 and 383338 bp of pAMI4 in a location which is neutral for maintenance functions of the megaplasmid. *P. aminophilus* pAMI4TcΔ*3parS* was constructed based on *P. aminophilus* pAMI4Tc by deleting the whole *3parS* region, between 424710 and 426551 bp of pAMI4, and inserting a kanamycin resistance gene (*aph*) in its place. *P. aminophilus* UW100 was constructed by removing pAMI4 from *P. aminophilus* JCM 7686 by incompatibility with *3parS* delivered on pBBR1MCS-5 and subsequent loss of the vector by cultivation without the antibiotic pressure.

### Plasmid used in this study.

pBBR1MCS-5 (28) was used as a vector to introduce *3parS* (amplified pAMI4 fragment between 424627 and 426685 bp) to *P. aminophilus*. pBBR1MCS-3 (28) was used as a source of the *tetC* gene. pRK2013 (29) was used as a helper plasmid for triparental mating. pABW3 was used as an unstable vector for stability assays in *P. aminophilus* and *P. aminovorans*. Its four derivatives constructed for the stability assays carried (i) *repAB*_pAMI4_ (amplified pAMI4 fragment between 1312 and 3696 bp), (ii) *repAB*_pAMI4_ with *parS_BC_* (amplified pAMI4 fragment between 1184 and 3696 bp), (iii) *repAB*_pAMI4_ ([same as in (i)] and *3parS* (amplified pAMI4 fragment between 424627 and 426685 bp), and (iv) *repAB*_pAMI4_ with *parS_BC_* [as in (ii)] and *3parS* [as in (iii)]. pDS132 (*ori* R6K) (30) was used as a suicide plasmid with counterselection gene (*sacB*) for gene replacement in *P. aminophilus*. Its derivatives were used to insert the *tetC* gene into pAMI4 and to delete the *3parS* locus. pDYI-Km (31) was used as a source of the *aph* gene. pET28b(+) (EMD Biosciences) was used as a plasmid for overexpression of the 6×His-RepB_pAMI4_ protein (*repB*_pAMI4_ gene was amplified with primers 5′-CATAAGCTTTCACCCATCGGACTTAGCCTTG-3′ and 5′-GGTCATATGGCACGCAAGGATCTTCTCAAAG-3′ and cloned into pET28b(+) using HindIII and NdeI). pUC18 (32) was used as a cloning vector of artificial DNA fragments carrying single *parS* sequences and the *3parS* locus. pJC43 was used for expression of a GFP-RepB_pAMI4_ fusion protein from P*_tauAB_* promoter positively induced by taurine in the presence of TauR (33, 34). pZE1-GFP (35) was a source of the *gfp* gene.

### Plasmid stability assays.

Stability of pABW3 and pAMI4 derivatives was tested by monitoring the percentage of antibiotic resistant bacteria (kanamycin for pABW3 derivatives and tetracycline for pAMI4 derivatives) in populations grown in liquid cultures without antibiotics, as described previously (36). Cultures of *Paracoccus* spp. for the stability assays (20 mL in 100 mL flasks) were refreshed at 24 h intervals (dilution 1:1000) and continued for up to approximately 200 generations (ca. 400 h, 2 h/generation). In selected time points, the cultures were plated on LA in appropriate dilutions to obtain single colonies, which were tested for the antibiotic resistance by replicas on LA and LA with kanamycin or tetracycline. All stability assays were performed in triplicate.

### Overexpression and purification of recombinant 6×His-RepB_pAMI4_ protein.

The *repB* gene of pAMI4 was amplified by PCR and cloned into expression vector pET28b(+), to obtain pET28b+repB. RepB_pAMI4_ with an N-terminal 6×His tag was overexpressed in E. coli BL21(DE3)(pET28b+repB) and purified as described previously (37) with some modifications. A 1- L culture of the expression strain was grown to mid-log phase and production of the 6×His-RepB_pAMI4_ protein was induced by adding isopropyl β-D-1-thiogalactopyranoside (IPTG) to a final concentration of 0.4 mM. After further incubation for 5 h at 28°C the cells were harvested by centrifugation and lysed in 15 mL of lysis buffer (50 mM sodium phosphate pH 8.0, 300 mM NaCl, 10 mM imidazole and 300 μL of 100 mg/mL lysozyme, supplemented with 1 mM PMSF and Protease Inhibitor Cocktail; Sigma). After sonication and centrifugation, the cleared lysate was incubated with 0.5 mL of Ni-NTA beads (Qiagen) for 30 min, with gentle shaking. The Ni-NTA resin was then given a series of washes with (i) 3 mL of W1 buffer (50 mM sodium phosphate pH 8.0, 300 mM NaCl, 10 mM imidazole), (ii) 2 mL of W2 buffer (50 mM sodium phosphate pH 8.0, 2 M NaCl, 10 mM imidazole), (iii) 16 mL of W3 buffer (50 mM sodium phosphate pH 8.0, 300 mM NaCl, 50 mM imidazole). The 6×His-tagged protein was eluted in 3 lots of 0.5 mL of elution buffer (50 mM sodium phosphate pH 8.0, 300 mM NaCl, 250 mM imidazole). The final concentration of the purified recombinant protein was estimated using the Bradford dye-binding method (38). Protein aliquots were frozen in liquid nitrogen and stored at −70°C.

### Production of fluorescein (FAM)-labeled DNA fragments.

The *3parS* DNA fragment of pAMI4 (519 bp) was amplified by PCR with primers 5′-ATCCCCGGGCTTATGGGATTCATCGCCGCTG-3′ and 5′-ATCCCCGGGCAAATCTGTCGCGAGCCAGC-3′. To create individual partitioning sites, pairs of complementary synthetic oligonucleotides containing single *parS* sequences (primers 5′-AATTACGCTGTTGACAGCTGTCAACGAAAG-3′ and 5′-GATCCTTTCGTTGACAGCTGTCAACAGCGT-3′ for *parS*_BC_; 5′-AATTCCGCTGTTGACAGCTGTCAACTCGTG-3′ and 5′-GATCCACGAGTTGACAGCTGTCAACAGCGG-3′ for *parS*_1_; 5′-AATTGACGAGTTGACAGCTGTCAACGAGGC-3′ and 5′-GATCGCCTCGTTGACAGCTGTCAACTCGTC-3′ for *parS*_2_; 5′-AATTGCTCGGTTGACAGCTGTCAACTCGGG-3′ and 5′-GATCCCCGAGTTGACAGCTGTCAACCGAGC-3′ for *parS*_3_) were mixed together at a molar ratio of 1:1. Diluted oligonucleotide mixtures (10 μmol of each) were incubated at 95°C for 10 min and then slowly cooled to room temperature. The annealed synthetic dsDNA fragments, as well as the *3parS* region amplicon were cloned into the SmaI site of pBGS18. The resulting plasmids were used as templates in PCR with standard primers M13pUCf (5′-CCAGTCACGACGTTGTAAAACG-3′) and FAM-labeled M13pUCrFAM (5′-AGCGGATAACAATTTCACACAGG-3′). The same primer pair was used for the amplification of a 136-bp DNA fragment of pBGS18, which served as a negative control.

### DNA binding assay.

DNA-protein complexes were detected using a gel electrophoresis mobility shift assay (EMSA). First, competitor DNA (salmon sperm DNA) was incubated with 0, 10, 20, 50, 100, 150 or 200 pM of purified 6×His-RepB_pAMI4_ protein for 10 min at room temperature in a 12 μL volume of binding buffer (40 mM Tris-HCl pH 8.0, 10 mM MgCl_2_, 1 mM dithiothreitol, 100 mM KCl, 10% BSA). Then, 1 pmol of each FAM-labeled DNA fragment was added to each variant (8 μL). Next, 4 μL of 50% glycerol were added to the reaction mixtures before loading on a gel. The complexes were separated by electrophoresis in 5% non-denaturing polyacrylamide (19:1 acrylamide:bisacrylamide) gels cast in 0.25 × TBE (22.5 mM Tris base, 22.5 mM boric acid, 0.5 mM EDTA, pH 8.0) and run in 0.5 × TBE. Fluorescent DNA-protein complexes were visualized with a GE Healthcare AI600 imager.

### Fluorescence microscopy.

Plasmid pJC43-GFP-RepB was constructed for taurine-dependent expression of a GFP-RepB_pAMI4_ fusion protein and introduced from E. coli into P. aminophilus JCM 7686R carrying pAMI4, pAMI4Tc or pAMI4TcΔ*parS* and strain UW100 lacking pAMI4. Overnight cultures of the strains carrying pJC43-GFP-RepB, grown on LB with gentamicin, were diluted 1:1000 in LB with gentamicin plus taurine (or without taurine for the negative control) and grown to mid-exponential phase (OD_600_ 0.2) prior to imaging. Cells were transferred to 1.5% agarose-padded slides containing LB medium with gentamicin and taurine (or without taurine for the negative control). A coverslip was placed on top of the agarose pad and sealed with a vaseline:lanolin:paraffin mix (ratio 1:1:1) to prevent pad evaporation. Slides were placed under a Zeiss ApoTome inverted wide-field microscope. Bacterial cells and the GFP-RepBpAMI4 signal were imaged in the brightfield and the green channel respectively using a Plan Apo 63× objective (numerical aperture = 1.4, +optovar 1.6×) and a Hamamatsu sCMOS ORCA-Flash 4.0 v3 (Institut Pasteur Imaging Facility Imagopole). Images were taken using a 600 msec exposure. For each experiment, randomly selected images of 100 cells were analyzed to track the localization of the GFP-RepB_pAMI4_ fusion using ImageJ Fiji software (NIH) (39). The GFP fluorescent signal was only observed where taurine was added to the culture, and not in the non-induced controls.

### Bioinformatic analyses.

Sequence similarity searches of the GenBank database were performed using online BLAST tools (https://blast.ncbi.nlm.nih.gov) (40). The GC profile of plasmid sequences was determined using Artemis software (41). Protein phylogenetic trees were generated using MEGA software (42–44) by the maximum likelihood method with 500 bootstrap replications or by the neighbor joining method with 1000 bootstrap replications (for the tree presented in Fig. 3, the neighbor joining method was selected for ease of presentation, as the maximum likelihood tree of the same set of sequences showed a more complicated topology for branches with poor statistical support. For comparison, the maximum likelihood tree is presented in Fig. S4 in the supplemental material. Putative *parS* sites within the *repABC* region (defined as the region including *repA*, *repB* and *repC* plus the adjacent intergenic regions) were identified as palindromic or almost palindromic (up to one nucleotide position disturbing the palindrome) sequences matching the *repABC parS* consensus (5′-GTTNNNNGCNNNNAAC-3′) (5). If such sequences were found in the *repABC* region, they were used to search the whole available plasmid sequence for additional putative copies of *parS*, with a tolerance of a one nucleotide change.
