# Supplementary material for: Differential Localization and Functional Specialization of parS Centromere-Like Sites in repABC Replicons of Alphaproteobacteria
Source: Appl Environ Microbiol. 2022 Apr 7;88(8):e00207-22. doi: 10.1128/aem.00207-22 (PMC9040568; doi:10.1128/aem.00207-22)
Supplement: Supplemental file 1 — Fig. S1 to S4. Download aem.00207-22-s0001.pdf, PDF file, 0.8 MB [file aem.00207-22-s0001.pdf]

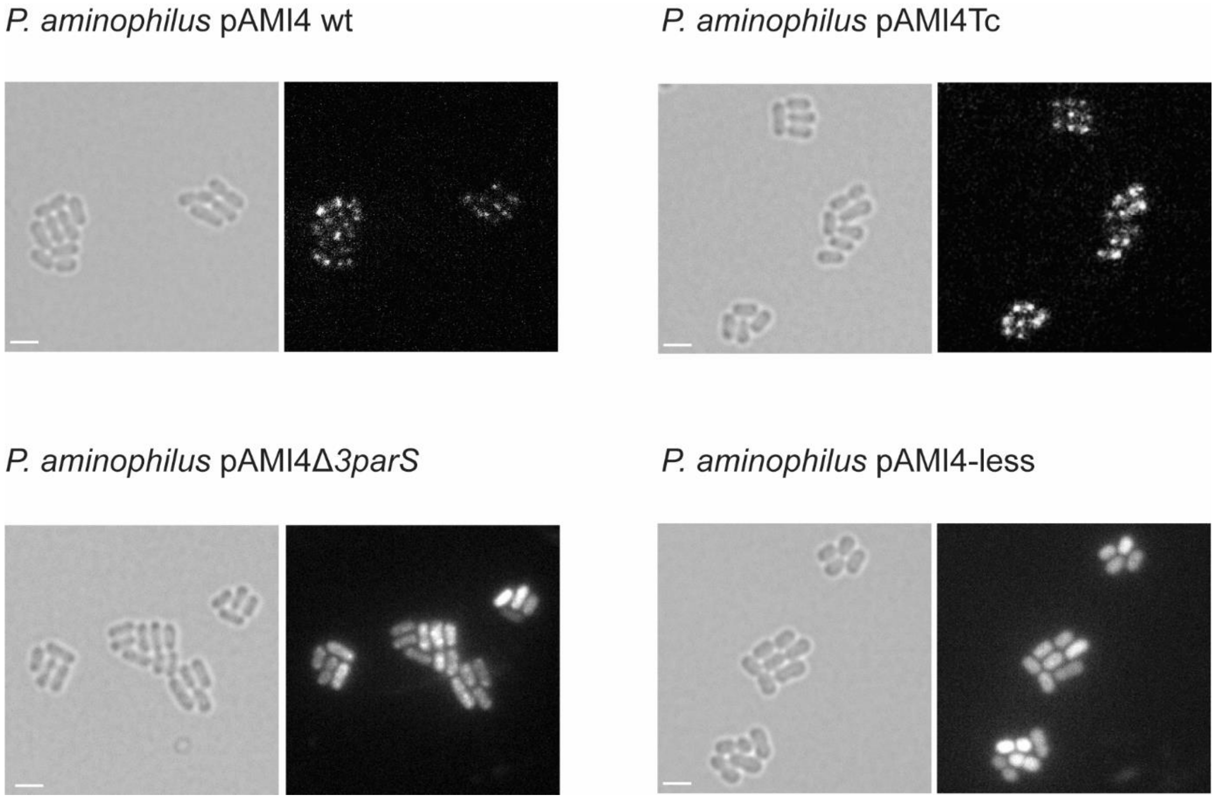

FIGURE S1. Expression and localization of GFP-RepBpAMI4 in the various *P. aminophilus* strains. Microscopy images of *P. aminophilus* cells (bright field image) expressing GFP-RepBpAMI4 (fluorescent image). All strains were cultured to mid-exponential phase in LB with gentamycin and taurine. Scale bar in all images is 2 microns.

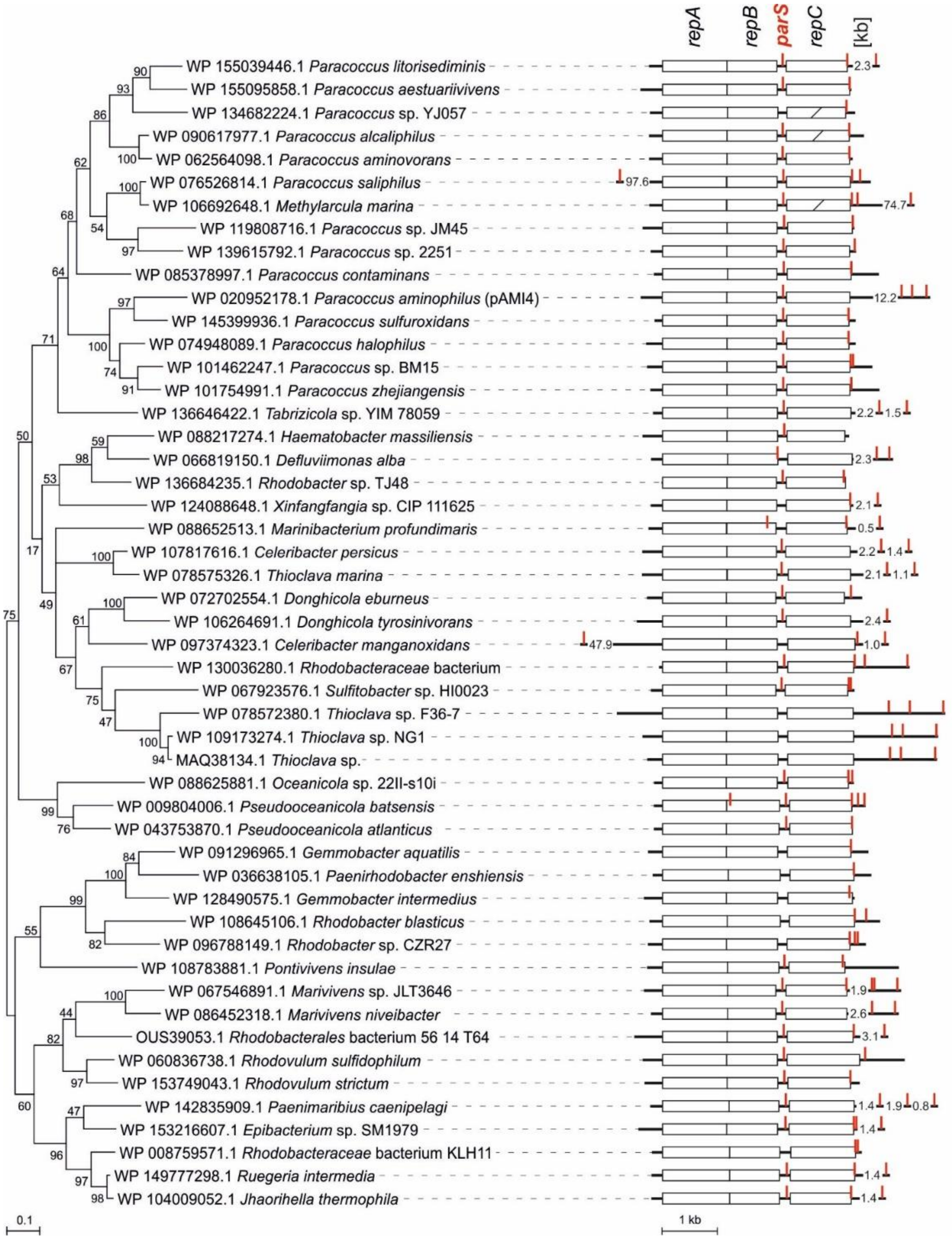

FIGURE S2. Distribution of the putative *parS* sequences within 50 *repABC* replicons of *Rhodobacterales* (replicons selected from database 2, Table S2; complete and partial plasmid sequences). In the left-hand panel, a maximum likehood tree of RepB protein sequences is presented. In the right-hand panel, the identified parS sequences are marked in red on diagrams of *repABC* modules that show the *repA*, *repB* and *repC* genes (rectangles) and intergenic regions upstream of *repA*, between *repA* and *repB*, between *repB* and *repC* and downstream of *repC* (bold lines). The intergenic/intragenic localization of putative *parS* sequences located outside the *repABC* modules (behind the numbers giving the distance from the *repABC* module in kb) is not indicated.

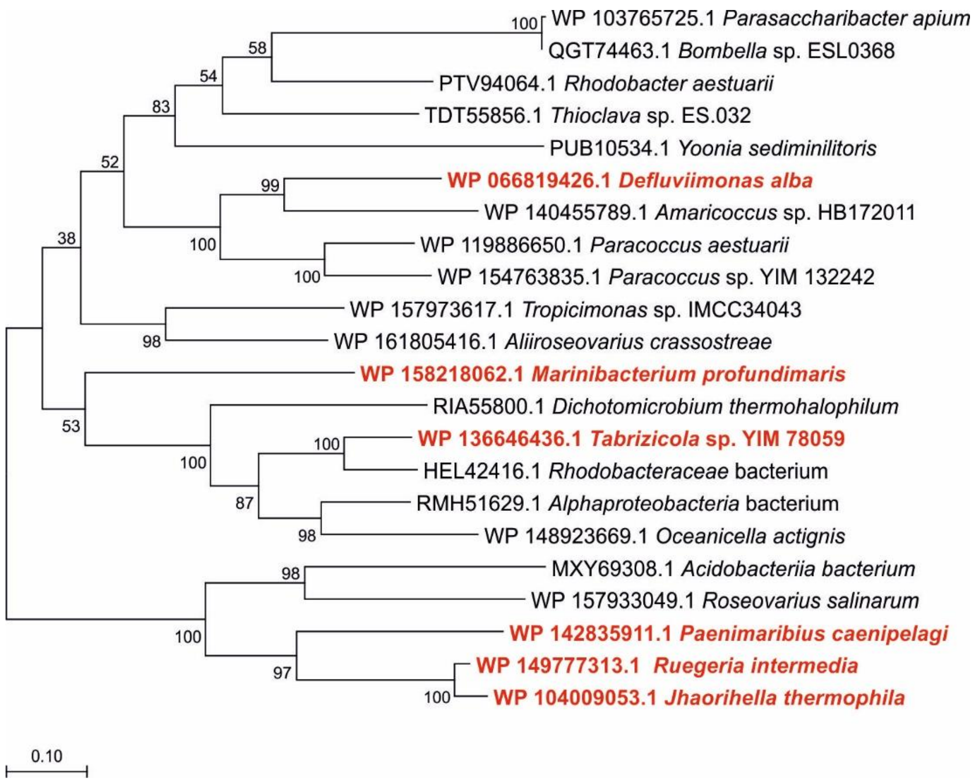

FIGURE S3. Maximum likehood tree of Cas3 proteins. The Cas3 proteins encoded directly downstream *repC* in the dataset 2 (Table S2) of the *repABC* replicon contigs are highlighted in red. Some additional related Cas3 protein sequences were used for the phylogenetic analysis (all non-highlighted), but none of them was encoded in the direct vicinity of *repABC* modules.

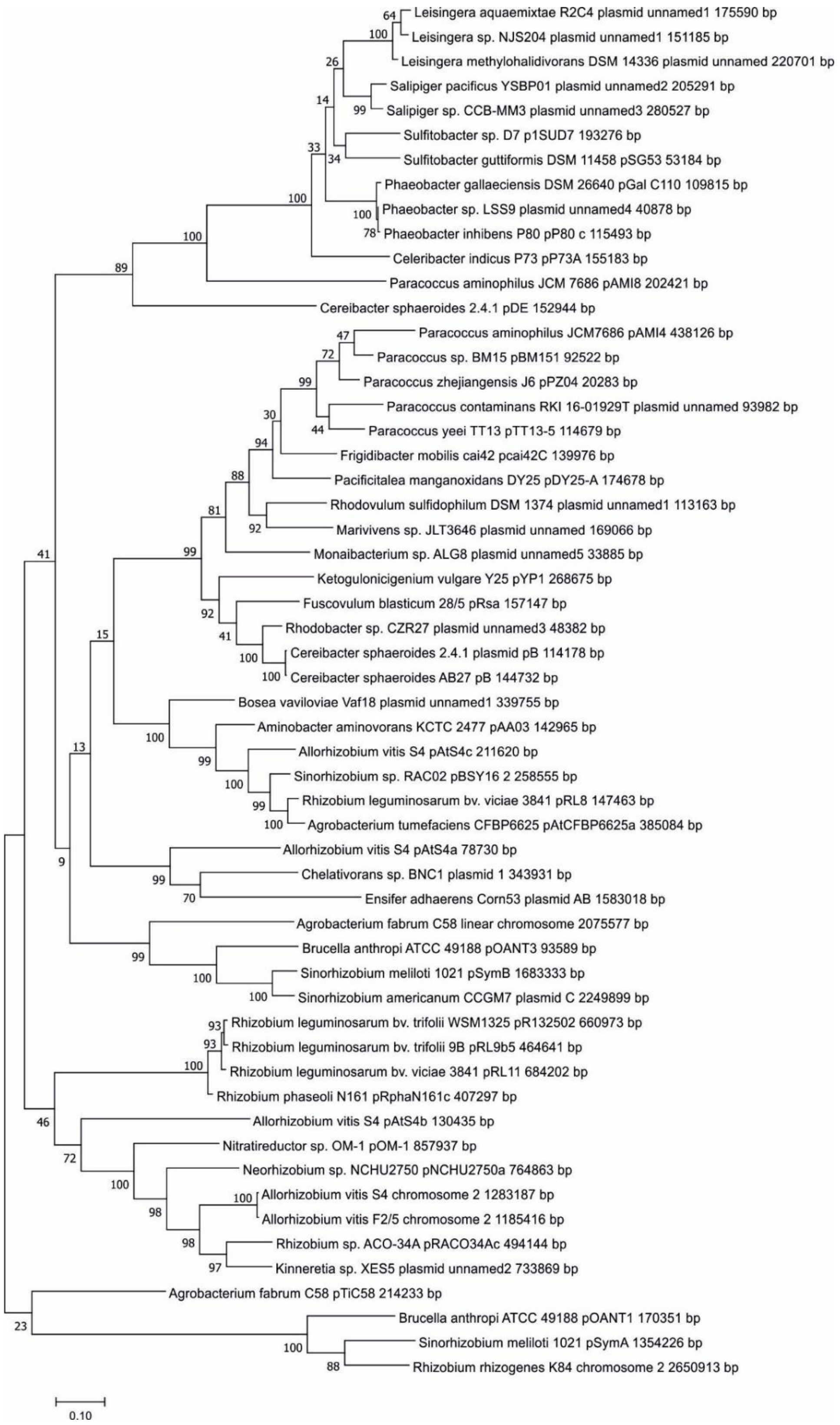

FIGURE S4. Maximum likehood tree of RepA-RepB concatenated protein sequences.
